# Atlanta Society of Medicine

**Published:** 1885-09

**Authors:** 


### ATLANTA SOCIETY OF MEDICINE.

STATED MEETING, August 4, 1885.

Dr. W. S. Elkin in the chair.

Dr. Virgil O. Hardon reported

### A CASE OF PREMATURE SENILE ATROPHY OF THE UTERUS.

A. B., aged 36, widow, commenced to menstruate at 14 years of age, married at 16. At the age of 24 she had had two miscarriages and six children at full term, none of which were twins. During this period she menstruated but very few times, as her pregnancies followed each other very closely. Her last child was born when she was 24 years of age, soon after which her husband died. She weaned this child at six months, but her menses did not return, and for twelve years since that time she has seen no sign of them. There appears to have been some attempt at vicarious menstruation, as she has had bleeding from the gums at quite regular intervals during a part of the time, and for a little while she vomited blood quite regularly every month. These phenomena ceased five or six years ago. For the last three years she has suffered from attacks of watery diarrhœa, which have shown a certain amount of periodicity, coming on at intervals of from four to six weeks and lasting from five to ten days. She has had at times a moderate amount of œdema of the lower limbs, which has appeared and disappeared without reference to treatment.

Her present condition is decidedly anæmic. Pulse 64—feeble. Temperature normal. Marked pallor of the face and lips. No

œdema at present. Heart sounds feeble, but otherwise normal. Auscultation shows no signs of pulmonary disease. Urine, specific gravity 1012, no albumen, no sugar. Area of hepatic dullness, normal ; no jaundice at any time. Appetite moderately good; digestion fair.

An examination per vaginam showed the cervix uteri to have almost entirely disappeared. It did not project into the vagina at all. The body of the organ was about one-half the normal size. Depth from external os to fundus, 1 1-4 inches. Ovaries could not be found. No tenderness in or around the uterus. No leucorrhœa.

Graily Hewitt describes this condition under the name of "premature senile atrophy," and gives the following brief list of symptoms : "The uterus affected with atrophy of the character alluded to is universally small, the cervix participates in the change, the vaginal portion becomes shorter, and the os uteri smaller. The tissues of the organ become somewhat harder."*

Sir Jas Y. Simpson attributes such cases to "superinvolution" after delivery.† The frequent pregnancies in the case reported above, and the disappearance of the menses after the last labor, might seem to lend some support to this theory. But Emmet cites a case in which atrophy occurred in a young unmarried woman where the theory of "superinvolution" would of course be untenable. He says: "Atrophy of the uterus is often found in connection with amenorrhœa, not as the cause, but as an effect of a common cause. I have frequently observed atrophy taking place after the amenorrhœa had existed for some time."‡

My own opinion is that atrophy of the uterus is dependent upon a cessation of ovarian activity, and the cause of this cessation may be found in some defect of innervation. In the majority of cases the amenorrhœa may be traced to some strong mental or nervous impression (in this case the death of a husband), which, by a sudden shock, affects the ganglia, from which the

*Diseases of Women, New York, 1883 Vol. I, p 124.

†Clinical Lecture on Amenorrhœa, *Medical Times and Gazette*, 1861.

‡ Principles and Practice of Gynæcology. New York, 1884, p. 165.

ovaries derive the nerve force necessary to maintain them in a state of health. Emmet's observation that the amenorrhœa frequently precedes the atrophy for sometime corroborates this view.

In regard to the age at which menstruation ceased in this case, I find but two instances recorded of its disappearance at an earlier period. These two are by Brierre de Boismont, in which the menopause occurred at the age of 21.* Tilt reports one at 27.† Graily Hewitt one at 30.‡ Statistics on this point seem to be somewhat meagre. Most modern authors throw no new light upon the subject, but content themselves with quoting Brierre de Boismont's tables.

An interesting feature of this case is the repeated attempt to replace the suppressed menses by vicarious discharges from various parts of the body, taking the forms at different times of bleeding from the gums, hæmatemesis and serous diarrhœa. Instances of vicarious menstruation are by no means uncommon, but I have never seen or read of a case where it occurred under three different forms in the same subject.

The treatment in this case was directed only to the general condition of the patient. Any attempt to restore the menstrual function would be entirely useless after the lapse of twelve years. In the early stages of the affection, electricity might properly have been resorted to as a stimulant to the sympathetic ganglia, as it has proved successful in some cases.

Dr. Noble stated that, in cases of senile atrophy of the uterus, he had used the interrupted galvanic current with the result of reestablishing the functions of that organ.

Dr. Woodward reported a case in which ergot failed to produce uterine contractions. Mrs. A., age 21 years—uniparous menorrhagia—following probable abortion. Prescribed first, acid sulp. aromat ʒii., ext. ergot, fl. (Squib) ʒ vi. Sig. 1 ʒss. every two hours. Hemorrhage having increased, I gave P. D. & Co.'s nor-

---

* De la Menstruation dans ses rapports Physiologiques et Pathologiques Paris, 1842.

† On Uterine and Ovarian Inflammation, p 54.

‡ Opuscit, Vol. I., p. 57.

mal liquid ergot ʒii every half hour; two doses producing no effect in the uterus I gave a hypodermic of squibbs' ergotin of gr. v. These doses were followed by the cerebral physiological effect, but there were no uterine contractions. The hemorrhage was stopped by vaginal injections of hot water.

---

AN ILLUSTRIOUS "TEN-YEAR MAN."—At Carbondale, Illinois, there once lived a pretentious old quack—"a ten-year man"—by the name of O'Haven, or, if not O'Haven, a name the spelling of which began in the same way. One day he lounged into a store where there were several of the more reputable physicians of the town and a literary man. The literary man knew of the ignorance of the quack and concluded to expose it, and to do so inveigled the pretender into a scientific conversation. Turning to one of the more reputable physicians present, our literary friend, with a significant wink, began:

"Dr. R., what do you think of the controversy in the *Lancet?*"

Dr. R.—"Well, the fact is, I haven't made up my mind about it."

Literary Man—"But, Doctor, don't you think it strange that so important a question as the position of the liver should not have been settled before this? I have always thought that there was no doubt but that the liver was situated above the diaphragm."

Dr. R. (taking the hint)—"Well, it's not so strange after all. I've seen cases in which it was hard to tell. I confess, however, I have been of the opinion that in most cases, at least, it was below the diaphragm."

L. M.—"The question is certainly an important one or the London *Lancet* would not spend so much time and space discussing it. Ah, there is Dr. O'Haven. Doctor, you have had great experience; what is your opinion on the question that appears to be vexing the professional mind?"

Dr. O'H. ("ten-year man")—"Well, sir, that is a question to which I have given a great deal of attention. From my long study and extensive observation, I have, however, been able to settle the matter beyond all dispute. The liver, sir, is situated *half above and half below the diaphragm!*"

This is a true story.—*Cincinnati Med. Journal.*

